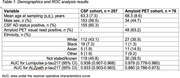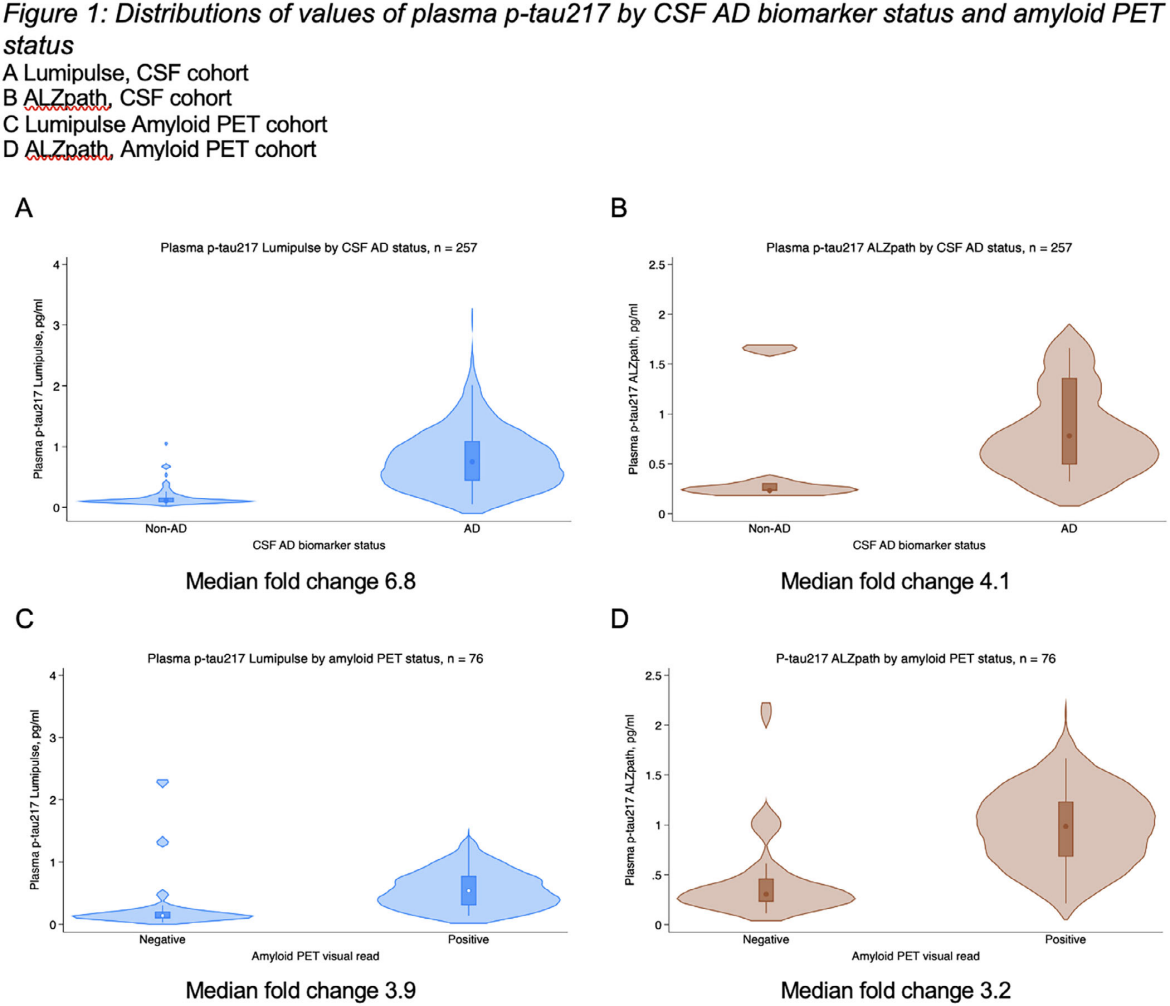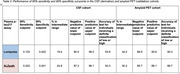# Alzheimer's Disease Diagnosis and Plasma Phospho‐tau217 (ADAPT) Stage 1

**DOI:** 10.1002/alz70856_100166

**Published:** 2025-12-24

**Authors:** Ashvini Keshavan, Katharine Wiltshire, Melanie Hart, Michael P Lunn, Paresh Malhotra, Jonathan M Schott

**Affiliations:** ^1^ Dementia Research Centre, UCL Queen Square Institute of Neurology, University College London, London, United Kingdom; ^2^ Dementia Research Centre, UCL Queen Square Institute of Neurology, London, London, United Kingdom; ^3^ Department of Neuroinflammation, UCL Queen Square Institute of Neurology, London, London, United Kingdom; ^4^ Neuroimmunology & CSF Laboratory, Queen Square, National Hospital for Neurology and Neurosurgery, London, London, United Kingdom; ^5^ Neuroimmunology & CSF Laboratory, Queen Square, National Hospital for Neurology and Neurosurgery, London, United Kingdom; ^6^ MRC Centre for Neuromuscular Diseases, UCL Queen Square Institute of Neurology, London, London, United Kingdom; ^7^ UK Dementia Research Institute Centre for Care Research and Technology, London, London, United Kingdom; ^8^ Imperial College London, Department of Brain Sciences, London, London, United Kingdom

## Abstract

**Background:**

ADAPT is a three‐stage UK multi‐centre study investigating the impact of plasma *p*‐tau217 on AD diagnosis, treatment, and quality of life and health economic outcomes. Stage 1 validated two commercially available plasma *p*‐tau217 assays against CSF AD biomarker status and derived cut‐points for clinical interpretation, which were then evaluated in an independent amyloid PET cohort.

**Method:**

Patients in the UCL CSF Cohort were classified as CSF AD biomarker positive if CSF Aβ42/Aβ40<=0.065 and *p*‐tau181>57 pg/mL (Lumipulse, Fujirebio). Patients in the Imperial Amyloid PET Cohort attended for clinical amyloid PET scans, categorized positive/negative by visual read. EDTA plasma samples from both cohorts were tested blind at a UKAS accredited clinical laboratory using the Lumipulse G (Fujirebio) and ALZpath Simoa (Quanterix) assays. We derived cut‐points for 95% sensitivity and 95% specificity from the CSF cohort, and evaluated the percentage of individuals assigned intermediate plasma *p*‐tau217 results, negative (NPV) and positive predictive value (PPV) of the tests. These cut‐points were then applied to the amyloid PET cohort to ascertain the same metrics in relation to amyloid PET status.

**Result:**

The CSF cohort (Table 1) comprised 257 individuals (mean age 63.3 years, standard deviation 7.3 years; 60% male, 60% CSF AD biomarker positive, median fold change for Lumipulse 6.8, ALZpath 4.1; Figure 1a and b). For Lumipulse, 95% sensitivity and 95% specificity cut‐points were 0.153 and 0.422 pg/ml, with NPV 91% and PPV 96% respectively; 19% of individuals had intermediate values. On applying these cut‐points to the amyloid PET cohort (*n* = 76, mean age 68.3 years, standard deviation 8.6 years; 45% male, 63 amyloid PET positive, median fold change for Lumipulse 3.9, ALZpath 3.2; Figure 1c and d), 34% had intermediate values, with NPV 89% and PPV 91%. For ALZpath, 25% of individuals had intermediate values in the CSF cohort and 30% in the amyloid PET cohort (Table 2).

**Conclusion:**

In both cohorts the accuracy of Lumipulse plasma *p*‐tau217 in those assigned either high or low values exceeded 90%, and NPV was greater than for ALZpath *p*‐tau217. The validated Lumipulse cut‐points will be implemented as part of a randomized controlled trial of result disclosure in community memory clinics.